# The simultaneous use of *Emotional suppression* and *Situation selection* to regulate emotions incrementally favors physiological responses

**DOI:** 10.1186/s40359-020-00495-1

**Published:** 2020-12-11

**Authors:** Simon Thuillard, Elise S. Dan-Glauser

**Affiliations:** 1 Institute of Psychology, University of Lausanne, Lausanne, Switzerland; 2 Department of Psychology, University of Fribourg, Fribourg, Switzerland

**Keywords:** Emotion regulation strategies, Emotion responses, Multiple strategy use, Strategy efficiency

## Abstract

**Background:**

Emotion regulation alters the trajectories of emotional responses and, when effective, transforms the emotional responses to help individuals adapt to their environment. Previous research has mainly focused on the efficiency of regulation strategies performed individually at a given time. Yet, in daily life, it is likely that several strategies are often combined. Thus, we question in this study the combinatorial efficiency of two emotion regulation strategies, *Situation selection* and *Emotional suppression*.

**Methods:**

In a within-subject design, sixty-five participants were asked to implement either no strategy, *Situation selection* only, *Emotional suppression* only, or both strategies together (four conditions) while looking at various emotionally charged images. Experience, expressivity, and physiological arousal were recorded throughout the viewing. Repeated-measures ANOVAs and corrected post-hoc tests were used for analyzing the data.

**Results:**

The results of the combined strategies showed that *Emotional suppression* canceled the beneficial impact of *Situation selection* on negative experience, while significantly increasing the impact on cardiac activity. The use of both strategies together had a greater effect on respiratory function with an enhanced decrease in respiratory rate and amplitude.

**Conclusions:**

The combinatorial effect of emotion regulation strategies is different according to the emotional response that the individual needs to regulate. The simultaneous use of *Situation selection* and *Emotional suppression* could be particularly beneficial to relieve physiological symptoms.

## Background

Emotion is a central aspect of psychological functioning, generally defined as a rapid psychological process leading to changes in behavior [1]. Emotion usually emerges following a situational trigger that is processed. It results in a set of experience, expression, and physiological arousal patterned responses, which help the individual adapt to the changing environment [2, 3].

Because of social display rules, personality, and individual preferences, a significant part of emerging emotional episodes are regulated [4]. Emotion regulation refers to “the processes by which individuals influence which emotions they have, when they have them, and how they experience and express these emotions” ([5, p. 275]). Emotion regulation alters the trajectories of the emotion responses and shapes the resultant affective reactions. Emotion regulation has been found to play a crucial role in healthy adaptation [6, 7] and efficient social functioning [8]. In contrast, difficulties in emotion regulation are associated with substance dependencies [9], anxiety and mood disorders [10–13], increased posttraumatic stress disorder [14], and personality related symptoms and pathologies [15–17]. Therefore, reaching functional emotion regulation is crucial in promoting individuals’ well-being and adaptation to the environment.

One influential model of emotion regulation is the Process Model of Emotion Regulation [18, 19]. It presents five typical strategies used at different times along the emotion generative process: *Situation selection* and *Situation modification*, which intervene before the emotional situation, *Distraction* and *Reappraisal*, which intervene simultaneously to the emotional situation, and *Suppression*, which modulates emotional responses once they have occurred. When questioning the relative efficiency of the different emotion regulation, strategies such as *Reappraisal* (i.e., changing the meaning of an emotional situation) or *Suppression* (i.e., modifying a behavior, mainly the emotion expressivity, to hide its manifestation) have drawn considerable attention. However, other strategies, such as *Situation selection,* have unfortunately received less consideration.

*Situation selection* “involves taking actions that make it more (or less) likely that we will end up in a situation we expect will give rise to desirable (or undesirable) emotions.” ([20] , p.11) (see also [21]). *Situation selection* was first considered as a unitary concept. However, it may operate through two different mechanisms [22]. The first one is related to the chosen situation: when comparatively evaluating the available options, people generally choose the one they expect to have the more pleasurable emotional outcomes [23, 24]. For example, if given the choice between calling a dear friend or doing some clean up, people will likely anticipate more positive emotions from the first situation. The consequence of *Situation selection* may here be due to the selected option being intrinsically more positive. In the second mechanism, *Situation selection* acts through the empowering action of making a decision about one’s own emotions. Thus, regardless of the options presented or selected, emotional responses to two identical situations might differ depending on whether the situation is chosen or not. For example, if on a chore list remains only “Paying the bills” (no other option to take instead), this activity will trigger more negative emotions than if, among the less pleasurable duties to perform, we select (*Situation selection*) “Paying the bills” instead of something else. This would be a consequence of *Situation selection* itself rather than a consequence of the intrinsic emotional value of the situation. A recent study in our lab examined this question by using a special within-subject protocol contrasting reactions to identical emotional stimuli under two different conditions: a) when a stimulation was chosen, and b) when the same stimulation was presented without any prior choice procedure [22]. Results showed that having the choice in negative situations decreased negative experience, skin conductance, and respiration reactivity, while enhancing expressivity and cardiovascular reactivity. In contrast, choosing a positive image before seeing it did not affect its positive value.

In the process model of emotion regulation, *Situation selection* operates before the situation occurs, allowing other emotions regulation strategies to be used together with *Situation selection*. In everyday life, individuals have at their disposal a wide range of emotion regulation strategies and it is likely that individuals combine them to cope with emotions. Indeed, 65% of participants watching a disgusting video clip are shown to use more than one emotion regulation strategy [25]. Moreover, across 10 emotional situations, between 78 and 92% of participants report using more than one emotion regulation strategy [26]. Finally, while looking to the extent to which participants combine instructed and uninstructed emotion regulation strategies, a study found that a significant proportion of participants reported using more than one emotion regulation strategy simultaneously [27].

Although the literature considers the combined use of emotion regulation strategies [28], there is still a lack of evidence of the potential interaction effect of multiple emotion regulation strategies implemented simultaneously. So far, research mainly focused on how much respondents use different single strategies [29–31]. This question is also related to the field of regulation flexibility, which assumes that strategies can be used in a sequential order (see e.g., [28, 32]). Our research, however, focuses on combined use efficiency rather than on percentage of respective use. We thus investigated the specific combined impact of different emotion regulation strategies on emotion responses. This study targeted the following question: “How does using two emotion regulation strategies at the same time rather than each of them separately influence their efficiency?”

To answer this question, we needed to test a regulation strategy that could be attempted simultaneously (i.e., for the same emotional situation) with *Situation selection*. One strategy that met this requirement was *Suppression*. We choose it for several reasons. First, *Suppression* is a well-studied strategy, with substantial empirical background on its efficiency. However, it has only been studied individually, never simultaneously to another strategy. Second, it is classically categorized as a maladaptive strategy. Indeed, apart from the expressive response, this strategy hardly relieves the experiential and physiological components of emotion [33], and even shows a rebound effect in physiological arousal [34, 35]. This is in direct opposition to what has been found for *Situation selection*. The effects of *Suppression* on emotional responses are however probably more complex. For example, some studies have reported no effect of *Suppression* on negative subjective experience [36–38], whereas others reported reduced levels of negative emotional experience [39, 40]. These differences can be attributed to different emotion induction methods, categories of emotions induced, types of *Suppression*, personality, emotional competences, gender, or the parameters considered (see e.g., [41–46]). A previous research with a similar design and population as in the present study used emotional pictures designed to rapidly generate negative or positive emotions. Results showed that *Expressive suppression* triggered stronger transient negative experience, strongly reduced expressivity, stronger decrease in heart rate, as well as reduced respiratory rate and amplitude as compared to reactions in absence of regulatory instructions [47]. Since effects of physiological and expressive suppression significantly overlap [47], and since we believe that *Suppression* does not only target expressivity, we decided to focus on general suppression, i.e., the complete emotional response suppression; whether expressive, experiential or physiological [48]. We named this strategy *Emotional suppression*.

In the present study, we investigated the single and combined effects of *Situation selection* and *Emotional suppression* in a within-subject design with a picture viewing paradigm. A picture-viewing paradigm is a protocol that uses pictures representing scenes able to induce different emotions of different intensities [49]. Pictures are presented to participants for a few seconds and the viewing launches emotional reactions. Pictures usually come from validated databases and are known to validly and reproducibly induce desired emotional states [50] and outcome valence (positive or negative, [51, 52]). Pictures were presented to elicit emotional processes in four different conditions: a) *Unregulated*, where neither *Situation selection* nor *Emotional suppression* were instructed, b) *Situation selection* only, c) *Emotional suppression* only, and d) *Double regulation*, during which both *Situation selection* and *Emotional suppression* were attempted for the same emotional event. Hypotheses were as follows:

1. *Situation selection* only: We expected similar results as previously highlighted [22]. Namely, *Situation selection* should result in reduced negative experience, unchanged positive experience, unchanged positive and negative expressivity, enhanced orienting effect for positive viewing, reduced skin conductance level for negative viewing and a decreased respiratory rate. These last two effects should occur particularly at the end of the recording period.
2. *Emotional suppression* only: We expected *Emotional suppression* to have similar effects as those repeatedly found in the literature. Namely, it should yield unchanged positive and negative emotional experience, a decrease in positive and negative expressivity, as well as respiration rate and amplitude, and a greater decrease in heart rate.
3. Double regulation: The combined effect of performing both *Situation selection* and *Emotional suppression* is harder to predict due to the lack of literature. We expected that emotional experience might be unchanged. Although *Situation selection* reduces negative experience, this effect could be canceled by *Emotional suppression,* which regularly fails to change experience. At the expressivity level, we expected reduction of positive and negative responses. Indeed, *Emotional suppression* is easy to achieve and shows marked effects on facial expression. There is no reason to believe performing *Situation selection* would interfere with such effect. Regarding physiological signals, we expected interaction, or at least additive effects for heart rate and respiratory rate. This would lead to a stronger decrease of these parameters than when each strategy is performed individually.

## Methods

### Participants

A power analysis with a power of 0.8 [53], with effect sizes derived from partial eta squares from previous similar studies (f = 0.20, see [22, 47, 48]), and α = 0.05, yielded a target sample size of 63 participants. To compensate for technical difficulties or participant drop-out, a sample size of 70 participants was targeted. We were able to recruit 69 participants, who came to the first session. Four of them were excluded, either because they did not meet one of the inclusion criteria (right-handedness, *N* = 1), because we experienced technical difficulties with our experiment (*N* = 2), or because of external interruption (fire alarm in the building, N = 1). Sixty-five participants thus fully participated in our study (32 males and 33 females). Participants were all either first year Psychology university students participating for course credits (*N* = 42), or other discipline first year students participating for the equivalent of $50 (*N* = 23). They were recruited during first year psychology courses or via advertisements posted on University information boards with a brief description of the project, without mention of emotion regulation. Participants’ age ranged between 18.4 and 32.4 years, with a mean of 21.1 years (SD = 2.37 years). Exclusion criteria were pregnancy, medication, and diagnosis of anxiety or mood disorder. Inclusion criteria were right-handedness, age between 18 and 45 years old, and general good health. Regarding health, participants were tested with the 12-Item Short-Form Health Survey (SF-12, [54]) and scored an average of 81.0% (SD = 9.1) of good health (100% being excellent mental, physical and social health). Since handedness may have an influence on emotion processing and physiological outputs [55, 56], all participants had to be right-handed. Handedness was measured thanks to the Edinburgh Handedness Inventory [57], which gives a score of − 100 for totally left-handed and + 100 for totally right-handed respondents. At this test, our sample had an average score of 74.08 (SD = 19.02).

### Design and conditions

The experiment consisted of 10 blocks of pictures, each testing one of the four conditions described below.

1. In the *Unregulated* condition (2 blocks), participants were not given the opportunity to choose the upcoming image and were not instructed to suppress; they simply watched the pictures as displayed.
2. In the *Situation selection only* condition (3 blocks), operationalized as previously described [22] in a picture-viewing paradigm, the participants had to choose the image that will be presented to them. The options were given with category words stating possible image content. The selected image was presented 3.5 s after the choice was made. No instruction of suppression was given.
3. The *Emotional suppression only* condition (2 blocks) was investigated as a variant of expressive suppression investigated in previous studies [36, 37] and validated as general suppression in previous work [48]. In this condition, participants were asked to suppress both their emotional expression and their physiological arousal. Participants in this condition were not given the choice about upcoming images. To simplify the text, this condition will be referred to as *Suppression*.
4. In the *Double regulation* condition (3 blocks), participants were given the choice of the upcoming image (thus performing *Situation selection)* and were instructed to suppress during the viewing of the image (thus performing *Suppression*).

**Fig. 1:**
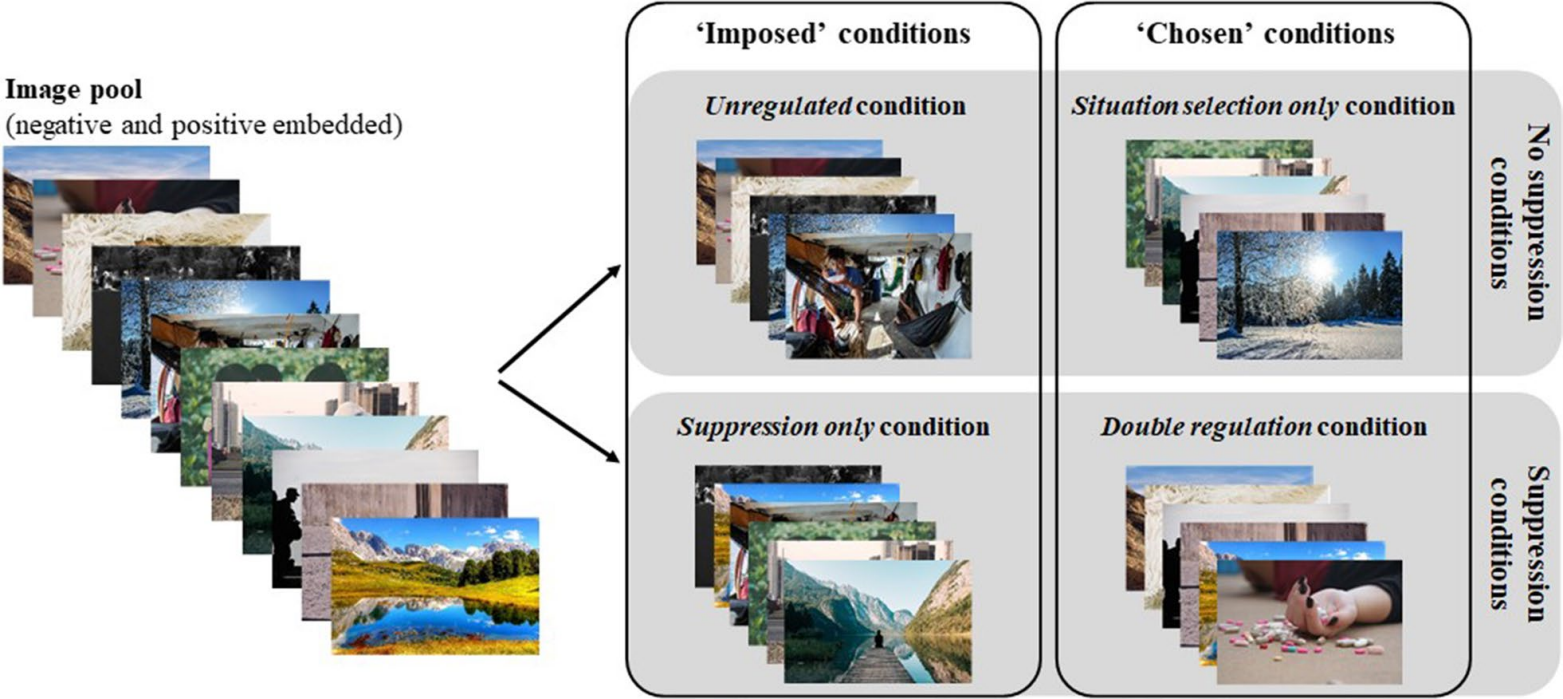
Illustration of the four conditions for testing the impact of *Situation selection*, *Suppression*, or both. Each condition included positive and negative trials. A posteriori, all trials of a particular valence were analyzed separately for each of the four conditions. Each participant had a different distribution of images in each of the conditions due to semi-random assignment of pictures to the conditions. Emotional responses were compiled for each of the eight trial types (valence × chosen/imposed × suppression/no suppression) and analyzed during the 8-s duration of each trial. Please note that the images shown here are for illustration purposes and are not part of the GAPED. These images were taken from www.pixabay.com and are free of copyright

These conditions are shown in Fig. 1. All the conditions in which choice was not given were called ‘Imposed’ conditions (*Unregulated* and *Emotional suppression* only): participants were not given the opportunity to choose and were imposed to watch emotionally relevant negative and positive pictures. All the conditions in which a choice was given were called ‘Chosen’ conditions (*Situation selection* only and *Double regulation* condition): in these conditions, participants choose the upcoming emotional picture.

The number of stimuli per block were arranged such that each block had approximately the same duration. There were 24 trials for the ‘Imposed’ condition blocks and 16 for the ‘Chosen’ conditions blocks. The block presentation was semi-randomized to control for habituation and order effects, only restricting the randomness by prohibiting the presentation of the same condition in consecutive blocks.

Before the start of each procedure, half of the stimuli were randomly assigned to the *Unregulated* and *Situation selection* conditions, and the other half to the *Suppression* and *Double regulation* conditions. Each of the stimuli was shown twice to each participant: once in the corresponding ‘Chosen’ condition, and once in the corresponding ‘Imposed’ condition. The stimuli that were not chosen in the ‘Chosen’ conditions were not considered for analyses in the ‘Imposed’ conditions. This allowed to equate the content of the stimuli between conditions for each participant, and to attribute the differences to our manipulation, rather than to the intrinsic features of the stimuli.

Finally, the timing of emotional response development is important in describing emotional arousal unfolding [58, 59], particularly in response to pictures [60, 61], and when considering emotion regulation impact [22, 47]. We therefore also included a time factor in our design, as it may provide more information on the potential transient or delayed impact of the respective emotion regulation strategies.

### Stimuli

Eighty seven images were taken from the Geneva Affective Picture Database (GAPED, [62]), which gathers negative and positive stimuli that can be included in different categories with labelled content. Having an available label for each picture category is crucial for operationalizing *Situation selection*, as it allows participants to make a choice based on written descriptors of the upcoming situations. The images of the negative category consisted of four content types: spiders, snakes, animal mistreatment and human mistreatment. These exact words were used as labels to offer a choice between categories in the ‘Chosen’ conditions. The positive images category also included four types of content: landscapes, human babies, mammals (usually offspring), and sport (inspirational) pictures. Since examples of the latter type of content are rare in the GAPED, we added nine pictures of sport/inspirational images from the International Affective Picture System (IAPS, [63]). The labels used to offer the choice for positive categories were “Landscape”, “Baby”, “Mammal”, and “Sport”. Of the final 96 pictures, 48 were negative and 48 were positive, with 12 pictures of each type of content. We presented emotional stimuli for eight seconds and examined participants’ emotional responses during this time-frame.

### Measures

We measured three emotional responses, corresponding to the three main emotional systems: emotional experience, expressivity, and physiological arousal [64–66]. For each, we derived parameters that reliably reflect rapid emotion emergence, and which proved to be sensitive to both our induction method and emotion regulation attempts.

#### Emotional experience

Participants used a rating slider to report their emotional experience (Variable Assessment Transducer, Biopac Systems, Inc., Goleta, CA, USA). Measures were taken continuously throughout the picture presentation. The slider was unipolar with negative reports on the left side and positive reports on the right side. The output voltage (0-9 V) was extracted as is and converted into a negative scale and a positive scale (see below the Data Reduction paragraph for more details on the data transformation for analyses).

#### Expressivity

Expressivity was assessed by using bipolar surface electromyography (EMG). Electrodes were standard 4 mm Ag/AgCl sensors. Three EMG sites were recorded. Because of its reliable link to negative expressivity, left *Corrugator Supercilii* [49, 67] was the first targeted site. The left *Zygomaticus Major,* and the left *Orbicularis Oculi* were the two sites chosen for positive expressivity. The zygomatic region is generally used to measure positive expressivity, which motivated our choice to record this site. However, the zygomatic is not a completely direct measure of positive expressivity [67]. We thus decided to add a supplementary channel and to target *Orbicularis Oculi,* a region that is a reliable readout of Duchenne’s smile [68]. The electrode placement followed recommendations of Fridlund and Cacioppo [69]. The skin was first gently rubbed with NuPrep® gel (Weaver and Cie). Excess gel was then removed with alcohol pads (Kendall Webcol® skin cleansing alcohol pads, Tyco healthcare). Finally, the electrodes were filled with Signagel® (Parker Laboratories, Inc).

#### Physiology

In order to tackle the different systems involved in autonomic reactivity, we measured cardiovascular, exocrine, and respiratory activities.

1. Electrocardiography (ECG): Three standard disposable pre-gelled Ag/AgCl electrodes were used for ECG recordings. One was placed approximately 5 cm below the lower rib on the left side of the abdomen. A second electrode was placed just below the right clavicle, along the mid-clavicular line. A third electrode, which served as a ground, was placed at the level of the C7 cervical vertebrae.
2. Electrodermal activity: Skin conductance level was recorded with two pre-gelled disposable Ag/AgCl sensors. They were placed on the thenar and hypothenar eminences of the non-dominant hand palm.
3. Respiration: Thoracic and abdominal respiration recordings were collected with two respiration belts. The abdominal belt was placed around the waist, whereas the thoracic belt was placed high on the chest.

All parameters were recorded and amplified with MP150 compatible modules from Biopac Systems (Goleta, CA, USA). All sensors were from the same company. All acquired channels were sampled at 1000 Hz.

### Procedure

Participation was divided into two sessions. In the first session, participants came in the laboratory and completed the SF-12 and the Edinburgh Handedness Inventory (see Participant section). They also completed other emotion-related questionnaires that served for another study. After checking right-handedness and absence of health-related issues, participants were invited to register for the second session.

About 11 days after the first session (average of 10.6 days, SD = 7.86), participants returned for the Emotion regulation task session. Upon arrival at the laboratory, participants were informed about the procedure of the experiment and prepared for the physiological recordings. All instructions were presented on screen. Participants were told that we were interested in people’s reactions to different scenes and that they would see different emotional images. The rating dial was introduced and we explained that the major task of the study was to report their feelings by moving the cursor while viewing each image. Some training trials were presented to familiarize participants with the rating system. They were then instructed about the *Situation selection* task. Instructions were as follow: “Sometimes in this session (in certain blocks), you will have the opportunity to choose yourself, from two options, the image you would like to see. Using the arrows on the keyboard, select the image category, then return to the slider and concentrate on your feelings to report them with the cursor.” The participants again performed a few training trials (with images that were not presented in the main session) in which they chose between two proposed options and reported their feelings when viewing the image. After this phase, participants were instructed about the *Suppression* task. Instructions were: “As you look at the images, you will realize that your face and body react to them. When you are asked to suppress your reactions, you should observe the image but NOT let the emotion affect your face and physiological reactions. Try to ensure that no one who would be looking at you, or at your physiological signals, can detect any changes in your face or body reactions.” Participants were again given some training trials for this task. Finally, they were informed that the experiment was divided into blocks, during which they would sometimes choose only, sometimes suppress only, sometimes both, and sometimes none of these, and that precise instructions about the task would be given before each block.

All participants saw blocks of images in the four conditions (see section above *Design and conditions*). Each participant completed the 10 blocks of trials, which were separated by a pause screen allowing participants to progress at their own pace from block to block. The 24 trials of the ‘Imposed’ blocks (both those with and without *Suppression*) were composed of 12 positive and 12 negative pictures, each with 3 images from each content category. ‘Chosen’ condition blocks (both those with and without *Suppression*) were generally composed of 8 positive and 8 negative pictures, each with 2 images from each content category. The last ‘Chosen’ block differed in the number of trials. This is due to the pairing procedure, the program exiting the last ‘Chosen’ condition block when it was no longer possible to couple unseen images of two different categories.

On average, participants performed 182 trials (range = 176–188), 48 in the *Unregulated* condition, 48 in the *Suppression only* condition, and on average 43 in each of the ‘Chosen’ conditions (*Situation Selection* and *Double regulation* conditions). All ‘Chosen’ blocks were thus of 16 trials except the last ‘Chosen’ block, averaging 6 trials. Each trial consisted of a blank screen (0.5 s), the choice screen (displayed until choice was made), a blank screen again (1.5 s), a fixation cross (1.5 s), a blank screen again (0.5 s) and the picture presentation (8 s). Under the ‘Imposed’ conditions (*Unregulated* and *Suppression only* conditions), the choice screen was replaced by the display of the category of picture to be presented (for 1 s). The computer session lasted approximately 55 min. The sensors were afterwards removed and participants were fully debriefed.

All participants gave written informed consent to participate in the study. The procedure was reviewed and authorized by the institutional and regional ethical committee (CER-VD, protocol 2015–00071), in accordance with the current national legal requirements (Ordinance on Human Research) and the latest version of the declaration of Helsinki.

### Data reduction

All data were processed with Acknowledge 4.4 (Biopac System, Goleta, CA, USA). Some channels were band-pass filtered to increase the signal-to-noise ratio (20–500 Hz for EMG, 0.5–35 Hz for ECG, and 0.05–1 Hz for respiration). Channels were then manually scanned for movement or electric interferences, which were corrected by signal interpolation. To assess the temporal dynamics of emotional response unfolding, the continuous parameters were segmented into 16 epochs of 0.5 s each. In addition to the image presentation duration of eight seconds, which was fully taken into account, a 3.5 s epoch was calculated for each trial and each parameter, spanning from 3.5 s before the picture presentation to the time of the picture onset. Thus, each trial had this reference period, which was used to normalize the response and obtain relative change in the parameter following each picture presentation.

#### Emotional experience

Ratings were exported to obtain average values for each epoch. The initial cursor position was used as a reference to calculate emotion intensity. Any value below this position was considered as a negative feeling and any value above as a positive feeling. On this basis, ratings were transformed into an emotion intensity scale. The output was extracted in percentages, representing the distance travelled by the cursor between its 0 point and its extreme values on either side. Data for each of the valence sides thus go from 0 = absence of emotional experience once confronted to the picture to 100 = extreme emotion intensity.

#### Expressivity

The EMG signals were rectified and smoothed (5 Hz) before being averaged for each epoch. Given the wide variability in the contraction capacity of each individual, each EMG time-frame value was then expressed as the percentage of contraction with respect to the corresponding trial antecedent level (voltage recorded for a given time-frame / voltage recorded during the 3.5 s preceding the trial * 100) [70, 71]. Negative expressivity was measured with the *Corrugator* site values, whereas Positive expressivity was measured with an average of the reactions measured on the *Zygomaticus* and *Orbicularis* sites, given the high correlation found for these parameters, r _(65)_=0.56, *p* < .001.

#### Physiology

Heart rate was calculated from the ECG channel by transforming the inter-beat interval (duration between successive R waves). Skin conductance level was exported as mean values for each epoch. Respiratory rate and respiratory amplitude were calculated for each epoch. The respiratory rate was obtained by converting the duration of the cycle intervals into a number of cycles per minute (c/min). The respiratory amplitude was interpolated by using the difference in volts between the point of maximum inspiration and the point of maximum expiration. Given the high correlations between thoracic and abdominal respiratory parameters (see “Result” section), the values of both sites were averaged. All response channel data were calculated as the change in activity with respect to each trial antecedent level.

### Data analyses

We first computed the descriptive values for each parameter in the *Unregulated* condition, as a manipulation check of reactivity. We contrasted the distribution of affective experience with a 0-centered distribution to confirm induction of presumed positive and negative experience.

Two steps of analyses were then successively conducted for each parameter:

- To investigate the potential interaction effect between the two regulation strategies, we first conducted, for each parameter, an ANOVA with three within-factors: Situation selection (2 levels: ‘Imposed’ vs. ‘Chosen’ conditions), Suppression (2 levels: no instructed Suppression vs. instructed Suppression) and Time (16 epochs). Since contrasting positive and negative trials was not part of our research question, but since previous research has shown different emotions [72–77] and emotion regulation patterns [78–80] for positive and negative responses, separate ANOVAs were performed for negative and positive trials. Except for the main effect of Time, which is relevant to the response dynamic but not to our research questions, all effects were of interest: (i) the main effect of Situation selection, for evaluating the general effect of choice as compared to the ‘Imposed’ conditions, (ii) the main effect of Suppression, for evaluating the effect of Suppression, irrespective of whether a choice is made, (iii) both two-way interactions Situation selection × Time and Suppression × Time, particularly interesting to evaluate the temporal dynamics of each emotion regulation strategy, (iv) the two-way interaction Situation selection × Suppression, to evaluate the potential combined effect of both strategies, irrespective of time, and finally, (v) the three-way interaction.
- To investigate further the effects found with the ANOVAs, we conducted analyses based on these initial results. We concentrated on the part of the viewing period for which the results of the analyses described above was significant. This strategy permitted us to focus on the period during which emotion regulation efficiency occured to further explore the differential efficiency of single vs. multiple strategy use when they are actually efficient. This strategy also decreased our Type II error risk by using a portion of the data that was not affected by general “reaction to picture” effect, common for all our conditions. We then re-ran the analyses on this portion of time, taking only the factors Situation selection and Suppression. Discriminating between additive or cumulative interaction effects in two-way ANOVAs is admittedly difficult [81]. Thus, we also conducted analyses observing one factor (four levels), directly contrasting the *Unregulated*, *Situation selection* only, *Suppression* only, and *Double regulation* conditions.

Greenhouse-Geisser corrections were applied where the assumption of sphericity was violated, and corrected degrees of freedom were reported in these cases. Effect sizes are reported using partial eta squares (η_p_^2^). *P*-values for interaction effects were corrected for multiple comparisons with the Holm-Bonferroni criterion. Threshold for significance was set to .05 (bilateral).

## Results

### Success of the emotion induction

Table 1 below shows the values of the parameters recorded over the entire viewing in the *Unregulated* condition, for both negative (left) and positive (right) viewing.

**Table 1:** Sample size (N), Mean, Standard Error of the Mean (SEM), and 95% Confidence Interval (CI) of Experience, Expressivity, and Physiological Arousal to Negative and Positive Images seen in the *Unregulated* condition

	N	Negative viewing			Positive viewing		
		Mean	SEM	CI (95%)	Mean	SEM	CI (95%)
Experience (/100)	65	41.25	2.21	[36.84; 45.67]	41.24	1.84	[37.57; 44.91]
Expressivity (EMG, %baseline)	65	150.76	8.42	[133.94; 167.58]	152.66	6	[40.67; 164.65]
Heart Rate (Δbpm)	65	−1.67	0.2	[−2.07; −1.27]	−1.47	0.22	[−1.90; − 1.04]
Skin Conductance Level (ΔμS)	56	−0.04	0.014	[− 0.07; 0.01]	− 0.07	0.01	[− 0.09; − 0.04]
Respiratory Rate (Δc/min)	65	− 0.07	0.05	[− 0.18; 0.03]	0.10	0.05	[0.00; 0.20]
Respiratory Amplitude (ΔmV)	65	−0.03	0.019	[−0.064; 0.014]	− 0.02	0.019	[− 0.056; 0.019]

*Note.* The experience scale ranges from 0 (*no emotion*) to + 100 (*extreme negative/positive experience*). Expressivity is expressed as percentages of trial baseline level. All other parameters are expressed as differences with trial baseline level to reflect parameter change after image presentation

On average, participants felt a significant increase of negative experience while confronted to negative images, mean = 41.25, t_(65)_=18.67, *p* < .001 (on a scale going from 0 to 100, with 100 being maximal negative emotion). Similarly, while confronted to positive pictures, they felt a significant positive experience, mean = 41.24, t_(65)_=22.44, *p* < .001 (on a scale going from 0 to 100, with 100 being maximal positive emotion), confirming successful induction of the desired emotional valence.

### Effect of *Situation selection* and *Emotional suppression* on emotional experience

**Fig. 2:**
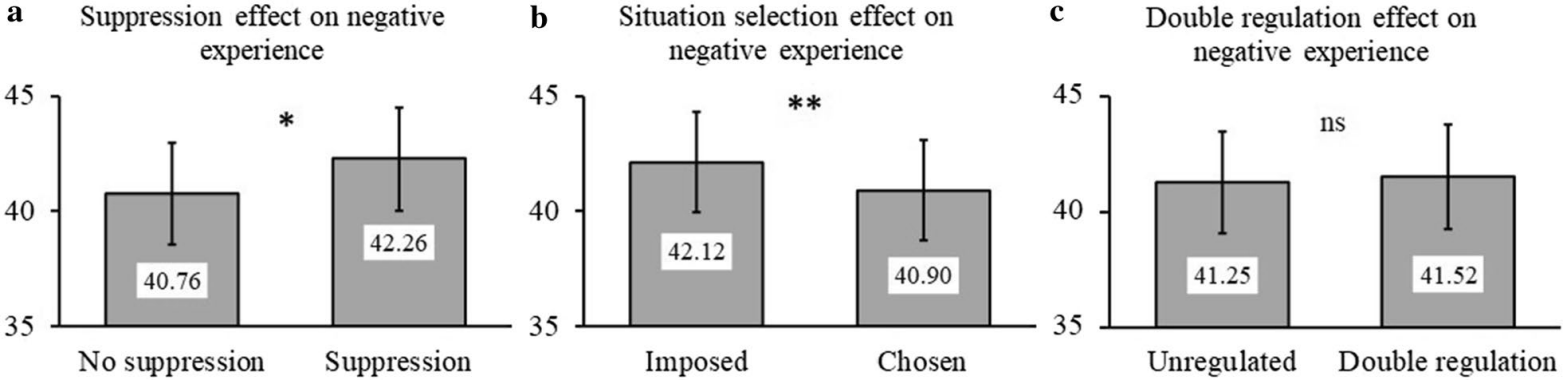
Effects of regulation on negative emotional experience. Are considered the main effect of Suppression (**a**), the main effect of Situation selection (**b**), or the contrast of the *Unregulated* condition with the *Double regulation* condition (**c**). The error bars are SEM. Ns = not significant, *p < .05, **p < .01

The three-way ANOVA Situation selection × Suppression × Time on negative trials yielded two main effects, that of Suppression, and Situation selection (see Table 2). Effect directions were opposite. Not using *Suppression* triggered a negative feeling of 40.76 points (Standard Error of Mean = SEM = 2.2), while attempting it increased the negative experience to 42.26 points (SEM =2.2). Conversely, without *Situation selection* negative experience was at 42.12 points (SEM = 2.2)*,* and at 40.90 points (SEM = 2.2) with it (see Fig. 2a and b). The lack of interaction effect (see Table 2) suggests that the two strategies might counteract each other. To further analyze their combinatorial effect, we directly contrasted the *Unregulated* condition with the *Double regulation* condition, with a two-way ANOVA Time (16 epoch) × Condition (4 conditions, see Fig. 1). Results showed neither an effect of the Condition, F_(1, 64)_=0.12, *p =* .74 (see Fig. 2c), nor an interaction of this factor with Time, F_(3,205)_ = 0.12, *p =* .95. Thus, it seems that the opposite effects of the two considered strategies cancel each other out when used simultaneously.

**Table 2:** Statistics for three-way ANOVA Situation Selection (SS) × Suppression (Sup) × Time (T) for Emotional Experience

	Negative viewing			Positive viewing		
	F (degrees of freedom)	***p***	η_**p**_^**2**^	F (degrees of freedom)	***p***	η_**p**_^**2**^
SS	9.71 (1, 64)	.003**	.13	0.82 (1, 64)	.37	–
Sup	4.09 (1, 64)	.047*	.06	0.70 (1, 64)	.41	–
SS × Sup	0.25 (1, 64)	.62	–	0.02 (1, 64)	.90	–
SS × T	2.33 (3, 198)	.07	–	2.09 (3, 161)	.11	–
Sup × T	1.39 (3, 188)	.25	–	5.45 (3, 189)	.001**	.08
SS × Sup × T	0.56 (3, 164)	.62	–	0.32 (3, 183)	.81	–

*Note.* *p < .05, **p < .01

When considering the impact of emotion regulation on positive experience, the three-way ANOVA Situation selection × Suppression × Time on positive trials yielded only one significant effect, that of the interaction between Time and Suppression (Table 2). Contrasts showed that the two groups of conditions (no Suppression conditions vs. use of Suppression) yielded different dynamics between 1 s and 2 s from the stimulation onset. During this time, positive experience increased less sharply without Suppression (average reached during this period = 23.23) than with it (average reached during this period = 26.13).

### Effect of *Situation selection* and *Emotional suppression* on emotional expressivity

The three-way ANOVA (Suppression × Situation selection × Time) on expressivity channels gave similar results for negative and positive trials. First, there was a main effect of Suppression for both negative and positive expressivity (see Table 3). These main effects showed that participants were very successful in performing *Suppression* as requested in the instruction. Indeed, this strategy triggered a decrease in the facial muscle activity from 151 to 107% of the trial baseline level for negative expressivity and from 156 to 103% of the trial baseline level for positive expressivity. For both valences, the ANOVAs showed significant interactions between Time and Suppression. Holm-Bonferroni corrected t-tests for each time frame showed that the differences in expressivity highlighted by the main effect did not happen immediately after the image onset but after 500 ms of viewing. No other effect was significant.

**Table 3:** Statistics for three-way ANOVA Situation Selection (SS) × Suppression (Sup) × Time (T) for Emotional Expression

	Negative viewing			Positive viewing		
	F (degrees of freedom)	***p***	η_**p**_^**2**^	F (degrees of freedom)	***p***	η_**p**_^**2**^
SS	0.05 (1,64)	.83	–	0.25 (1,64)	.62	–
Sup	32.95 (1,64)	<.001***	.34	81.15 (1,64)	<.001***	.56
SS × Sup	0.03 (1,64)	.85	–	3.92 (1,64)	.05	–
SS × T	0.31 (3, 221)	.85	–	2.39 (4, 259)	.05	–
Sup × T	9.33 (3, 211)	<.001***	.13	24.86 (3, 202)	<.001***	.28
SS × Sup × T	0.65 (4237)	.62	–	2.04 (5298)	.08	–

Note. ***p < .001

### Effect of *Situation selection* and *Emotional suppression* on physiological arousal

#### Heart rate

**Fig. 3:**
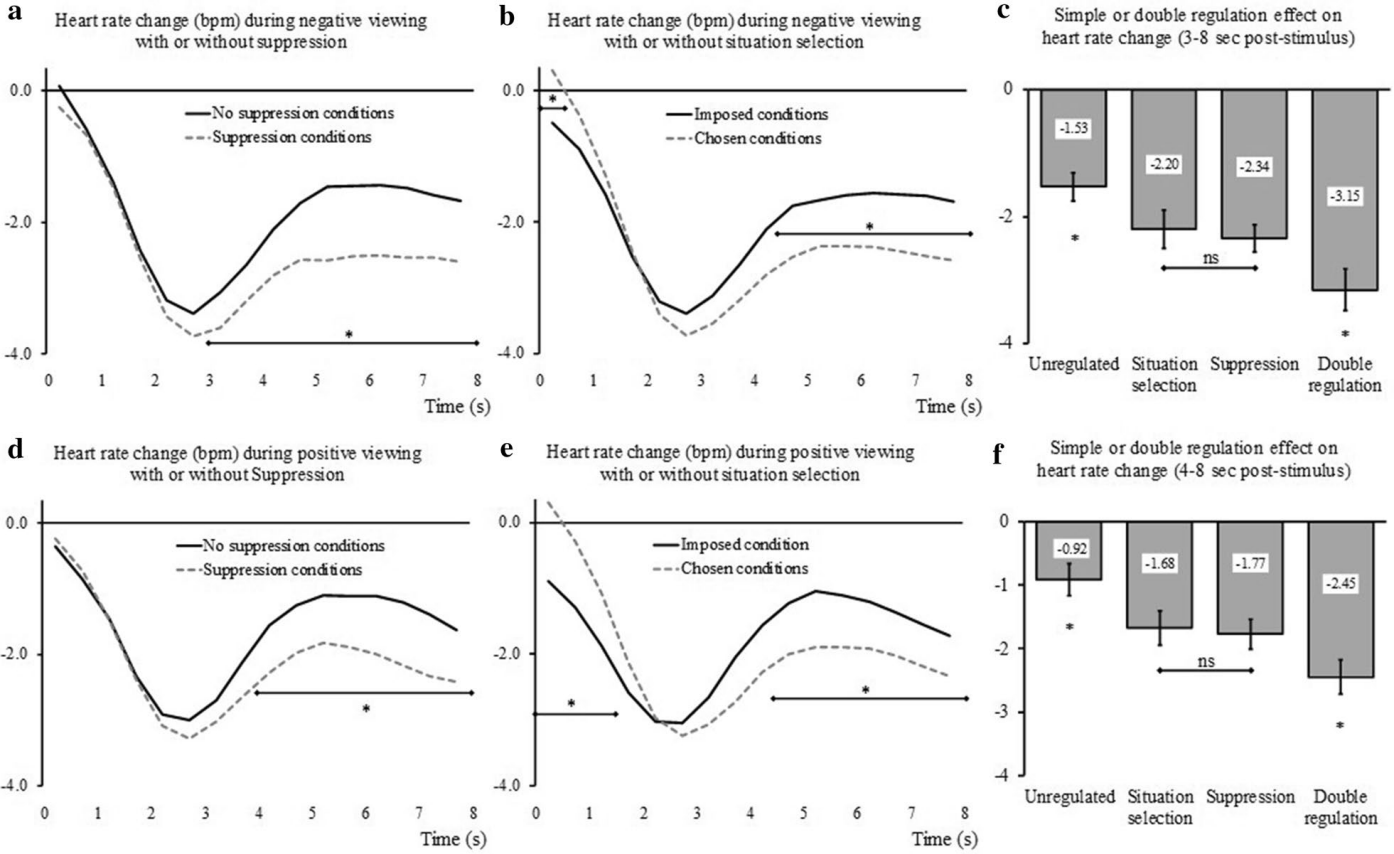
Effects of regulation on heart rate changes as compared to trial baseline level during negative (**a**, **b**, and **c**) and positive (**d**, **e**, and **f**) trials. Are considered the interaction between Time and Suppression (**a** and **d**), the interaction between Time and Situation selection (**b** and **e**), and the contrast between the *Unregulated* condition and the use of either one or the other strategy and to the *Double regulation* condition, in the period spanning between 3 s (**c**) or 4 s (**e**) and 8 s after image onset. The error bars are SEM. Ns = not significant, *p < .05, * in **c** and **f** represent significance of contrasts with each of the three other conditions

We first examined the impact of Suppression × Situation selection × Time on heart rate for negative viewing. The ANOVAs showed a main effect of Suppression (see Table 4), indicating that the decrease in heart rate observed during trials without Suppression (− 1.85 bpm) is less important than that observed with it (− 2.47 bpm). An interaction Time × Suppression was also noted (see Fig. 3a). Corrected contrasts showed that this effect occurred from 3 s after the image onset until the end of the recording period. Interestingly, a Time × Situation selection effect was also significant (see Fig. 3b), indicating an effect that was similar to that of Suppression. Performing the regulation (in this case *Situation selection*) triggered a stronger decrease in heart rate than without regulation, particularly from 4.5 s after the stimulus onset onward. No other effects were significant. As detailed in the “Data analyses” section above, we then contrasted, for the most relevant period (3–8 s after picture onset), the *Double regulation* condition with each strategy separately and the *Unregulated* condition. The test gave a significant effect of the Condition, F_(3,165)_ = 13.13, *p <* .001, η_p_^2^ = .17 and contrast results highlighted a cumulative impact of the two strategies (see Fig. 3c).

**Table 4:** Statistics for three-way ANOVA Situation Selection (SS) × Suppression (Sup) × Time (T) for Physiological Arousal

Parameter	Negative viewing			Positive viewing		
	F (degrees of freedom)	***p***	η_**p**_^**2**^	F (degrees of freedom)	***p***	η_**p**_^**2**^
*Heart Rate*						
SS	3.43 (1, 64)	.07	–	1.63 (1, 64)	.21	–
Sup	20.18 (1, 64)	<.001***	.24	11.16 (1, 64)	.001***	.15
SS × Sup	0.11 (1, 64)	.74	–	0.44 (1, 64)	.51	–
SS × T	14.57 (4, 234)	<.001***	.19	21.28 (3, 221)	<.001***	.25
Sup × T	6.81 (4, 245)	<.001***	.10	8.57 (4, 238)	<.001***	.12
SS × Sup × T	0.32 (4, 235)	.86	–	0.65 (4, 251)	.62	–
*Skin conductance*						
SS	0.41 (1, 55)	.53	–	0.01 (1, 55)	.91	–
Sup	2.95 (1, 55)	.09	–	0.03 (1, 55)	.85	–
SS × Sup	0.01 (1, 55)	.93	–	0.05 (1, 55)	.82	–
SS × T	10.36 (2, 90)	<.001***	.16	9.83 (2, 93)	<.001***	.15
Sup × T	0.30 (2, 110)	.74	–	2.81 (2, 110)	.06	–
SS × Sup × T	0.65 (2, 116)	.54	–	0.67 (3, 164)	.57	–
*Respiratory rate*						
SS	6.31 (1, 64)	.02*	.09	6.49 (1, 64)	.01*	.09
Sup	0.06 (1, 64)	.81	–	1.88 (1, 64)	.17	–
SS × Sup	2.38 (1, 64)	.12	–	2.47 (1, 64)	.12	–
SS × T	8.29 (2, 133)	<.001***	.12	18.50 (2, 157)	<.001***	.22
Sup × T	4.24 (2, 146)	.01*	.06	13.11 (2, 138)	<.001***	.17
SS × Sup × T	2.83 (2, 150)	.05	–	0.30 (3, 186)	.82	–
*Respiratory amplitude*						
SS	0.27 (1, 64)	.61	–	0.05 (1, 64)	.82	–
Sup	0.03 (1, 64)	.87	–	3.07 (1, 64)	.08	–
SS × Sup	3.79 (1, 64)	.06	–	5.32 (1, 64)	.024*	.08
SS × T	6.73 (3, 176)	<.001***	.10	4.47 (2, 154)	.009**	.07
Sup × T	2.57 (2, 107)	.09	–	8.53 (2, 126)	<.001***	.12
SS × Sup × T	1.17 (2, 158)	.32	–	0.81 (2, 159)	.47	–

Note. *p < .05, **p < .01, ***p < .001

We then examined the impact of Suppression × Situation selection × Time on heart rate for positive viewing. As with negative viewing, the ANOVA yielded a main effect of Suppression, again indicating that the decrease of heart rate observed during trials without *Suppression* (− 1.63 bpm) is less important than that observed with it (− 2.11 bpm). Similarly to the negative viewing results, a Time × Suppression interaction was also observed (see Table 4 and Fig. 3d), and corrected contrasts showed that this effect occurred from 4 s after the image onset until the end of the recording period. A Time × Situation selection effect was also significant (see Table 4 and Fig. 3e), indicating a similar effect as for Suppression. Performing *Situation selection* triggered a stronger decrease in heart rate than without it, in particular from 4.5 s after the stimulus onset onward. No other effects were significant. We then contrasted, for the most relevant period (4–8 s after picture onset), the double regulation effect with that of each strategy separately and that of the no regulation condition. The tests gave a significant effect of the Condition, F_(3,177)_ = 12.35, *p <* .001, η_p_^2^ = .16 (see Fig. 3f).

### Skin conductance level

**Fig. 4:**
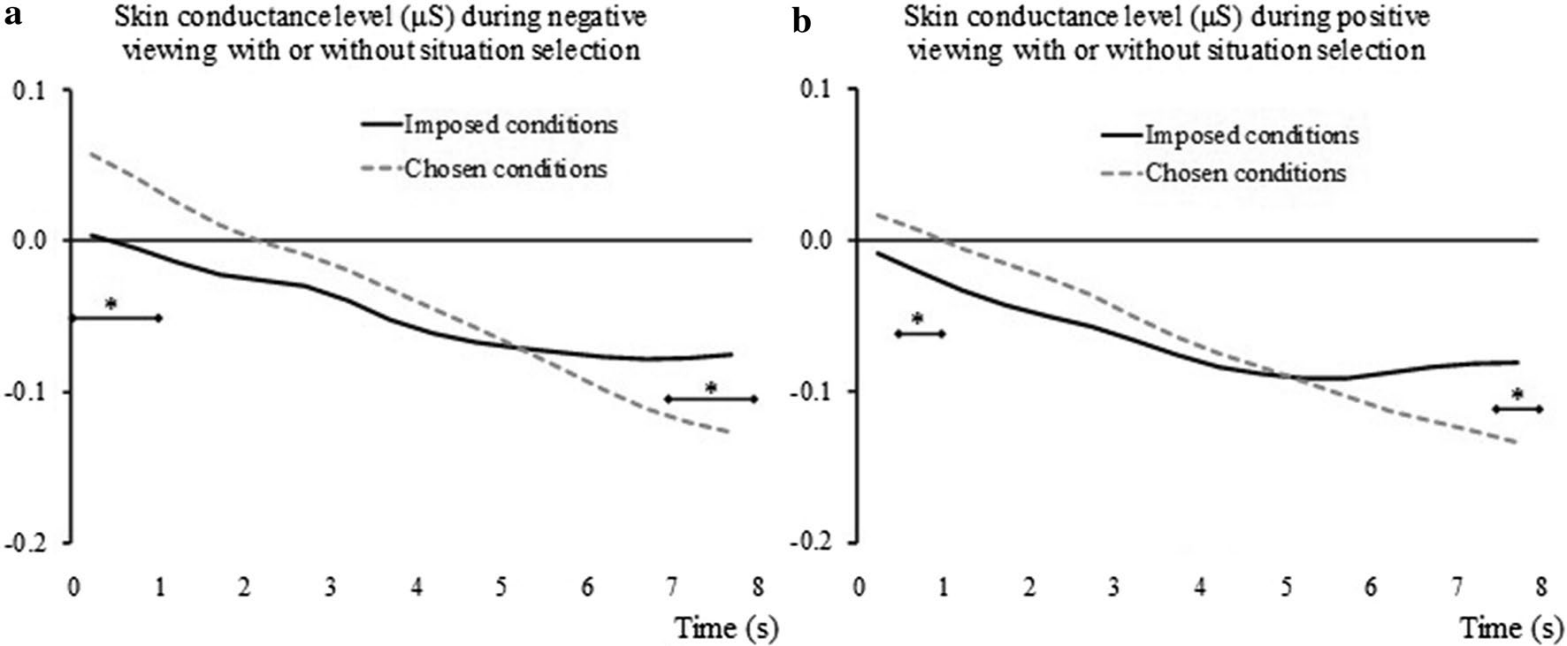
Effects of *Situation selection* on skin conductance level changes. Are depicted the reactions during the 8 s of the picture viewing for negative trials (**a**) and positive trial (**b**). *p < .05, corrected for multiple comparisons. Diamond ended lines indicate span of significant differences between the ‘Imposed’ conditions and the ‘Chosen’ conditions

We first examined the impact of Suppression × Situation selection × Time on skin conductance level change for negative viewing. Only one effect was significant, namely the interaction between Situation selection and Time (see Table 4). Contrasts indicated that, whereas the ‘Chosen’ conditions triggered a stronger increase in skin conductance level in the first second of the viewing, at the end of the image presentation (7–8 s after the image onset) the level was significantly lower than that observed in the ‘Imposed’ conditions (see Fig. 4a). No other effects in the ANOVA were significant.

While examining the ANOVA outcomes for positive viewing, we noticed a similar pattern of results. The Situation selection × Time interaction effect was significant, F _(2, 93)_=9.83, *p <* .001, η_p_^2^ = .15, while the other effects were not. Contrasts for each time frame on the ‘Chosen’ conditions, as compared to the ‘Imposed’ conditions showed that the effect seen in negative viewing was found also in positive viewing. However, it had shorter durations, the earlier effect spanning between 0.5 and 1 s after image onset, and the later effect occurring between 7.5 and 8 s after image onset (see Fig. 4b).

### Respiratory rate

**Fig. 5:**
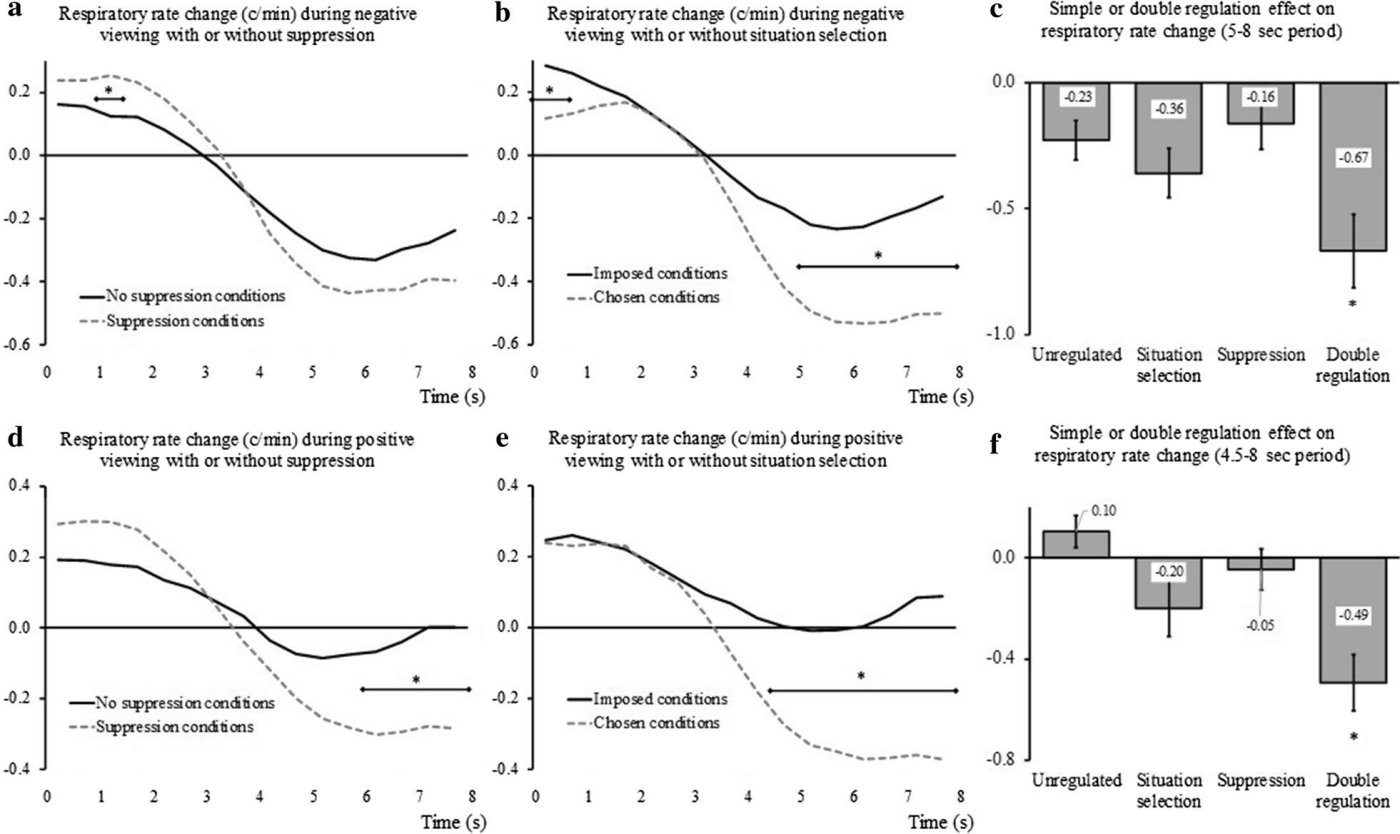
Effects of regulation on respiratory rate changes as compared to trial baseline level during negative (**a**, **b**, and **c**) and positive (**d**, **e**, and **f**) trials. Are considered the interaction between Time and Suppression (**a** and **d**), the interaction between Time and Situation selection (**b** and **e**), and the contrast between the *Unregulated* condition and the single strategy conditions (*Situation selection* or *Suppression*) as well as the *Double regulation* condition, in the period spanning between 5 s (**c**) or 4.5 s (**f**) and 8 s after image onset. The error bars are SEM. *p < .05, * in **c** and **f** represent significance of contrasts with each of the three other conditions

Since the correlation between thoracic and abdominal sites was of .79 (*p* < .001), measures at the two sites were averaged to form a single indicator of respiratory rate. When considering negative trials, the ANOVA performed on respiratory rate showed multiple significant effects. First, we found a significant main effect of Situation selection. Contrasts indicated that the decrease in respiratory rate that followed the presentation of the picture was greater under the ‘Chosen’ conditions (− 0.20 c/min) than under the ‘Imposed’ conditions (− 0.02 c/min). Second, we also found two significant interactions with Time, one with Suppression (see Fig. 5a), and one with Situation selection (see Fig. 5b). Despite overall similar shapes of the response profiles, contrasts indicated that the regulation conditions triggered a larger decrease in respiratory rate only when Situation selection was used, with an effect lasting from 5 s after the image presentation until the end of the recorded period. All the other effects of the ANOVA were not significant (see Table 4). Like for other parameters, we wondered about the effects taking place toward the end of the recording period, particularly regarding potential additive effect of the regulation strategies. Thus, we compared, in the most relevant period (5–8 s after picture onset) the *Double regulation* effect with that of each strategy separately and of the *Unregulated* condition. The tests gave a significant effect of the Condition, F_(3,177)_ = 5.91, *p =* .001, η_p_^2^ = .09 and contrasts showed a synergistic impact of the two strategies. The impact of *Situation selection* seemed potentiated by the combined use of *Suppression* (see Fig. 5c).

When considering positive trials, the ANOVA performed on respiratory rate showed multiple effects, similar to what was observed for the negative viewing. First, we found a significant main effect of Situation selection. Contrasts indicated that the decrease in respiratory rate under the ‘Chosen’ conditions (− 0.09 c/min) was significantly different from the opposed increase under the ‘Imposed’ conditions (0.11 c/min). Second, we also found here two significant interactions with Time: one with Suppression (see Fig. 5d), and one with Situation selection (see Fig. 5e). This time, the profiles for each regulation strategy showed similar significant contrasts. These indicated that the regulation condition triggered a larger decrease in respiratory rate towards the end of the recorded period (from 6 s post-stimulus onward for *Suppression* and from 4.5 s onward for *Situation selection*). All the other effects of the ANOVA were not significant (see Table 4). As for the other parameters, we wondered about the effects taking place toward the end of the recording period, particularly regarding potential additive double regulation effect. We compared, in the most relevant period (4.5–8 s after picture onset), the effect of *Double regulation* with that of each strategy separately and with that of the *Unregulated* condition. The tests gave a significant effect of the Condition, F_(3,167)_ = 10.70, *p <* .001, η_p_^2^ = .14 and contrasts suggested a incremental impact of the two strategies in reducing the respiratory rate (see Fig. 5f).

### Respiratory amplitude

**Fig. 6:**
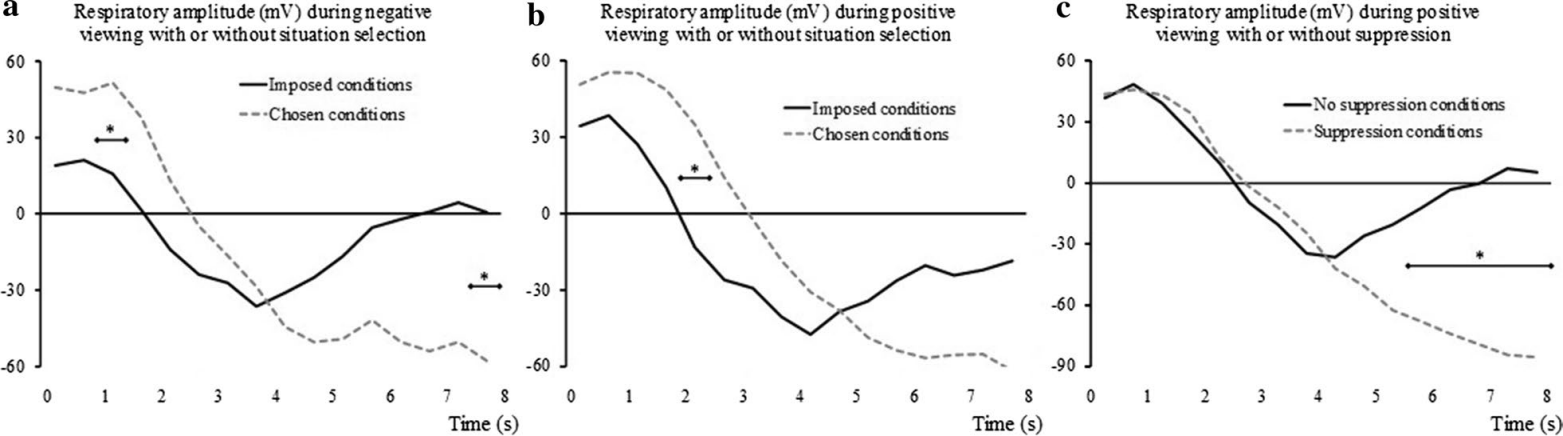
Effects of regulation on the respiratory amplitude change (in mV) as compared to trial baseline level. Are considered the interaction between Time and Situation selection during negative viewing (**a**), the interaction between Time and Situation selection during positive viewing (**b**), and the interaction between Time and Suppression during positive viewing (**c**). *p < .05, with Holm-Bonferroni corrections

**Fig. 7:**
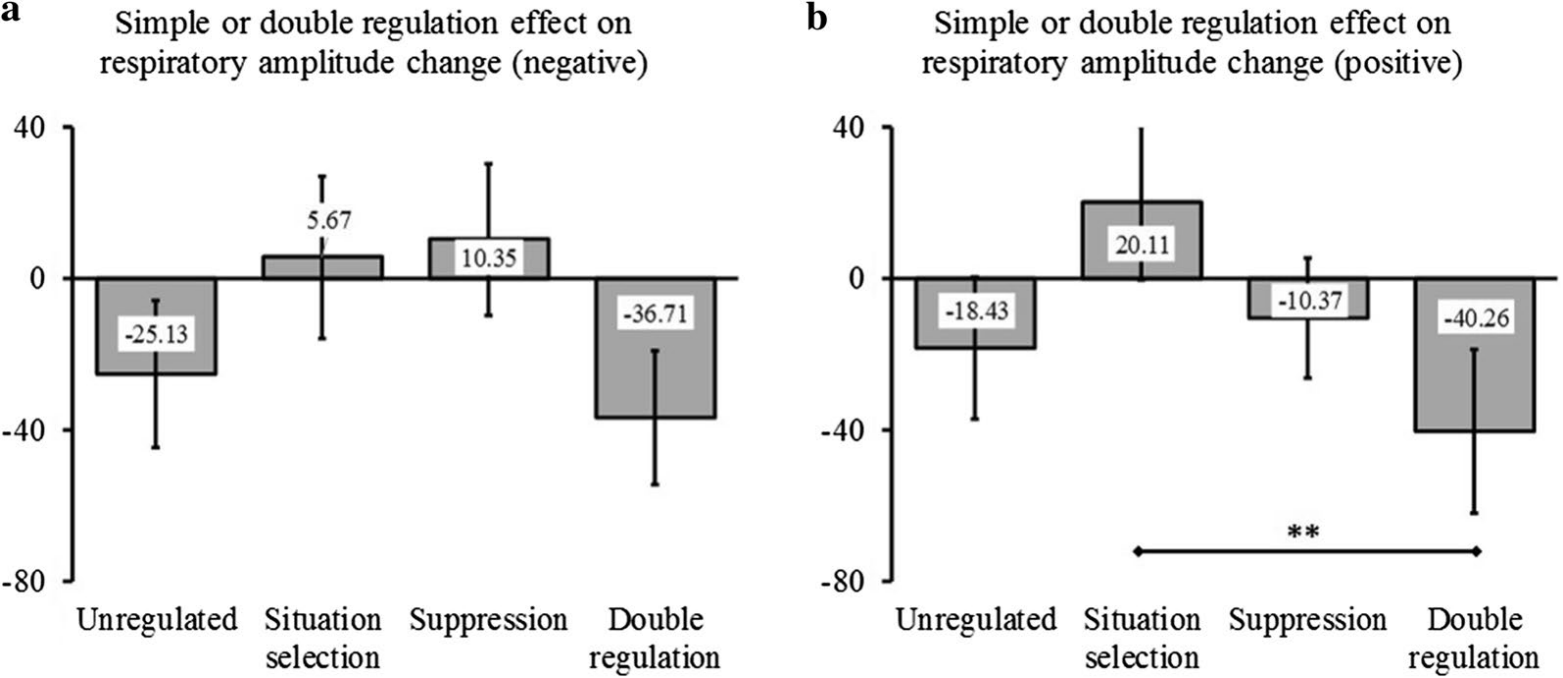
Effects of single and double regulation on changes in respiratory amplitude (in mV) as compared to trial baseline level. Are considered the negative viewing (**a**) and the positive viewing (**b**). Contrasts were calculated only for positive viewing (**b**) since the interaction Situation selection × Suppression was significant only for this valence. The error bars are SEM. **p < .01, with Holm-Bonferroni corrections

Since the correlation between thoracic and abdominal sites was of .36 (*p* = .004), measures at the two sites were averaged to form a single indicator of respiratory amplitude. When considering negative trials, the ANOVA performed on changes in respiratory amplitude only showed a significant interaction effect between Situation selection and Time (see Fig. 6a). No other effects were significant. Given the marginal significance of the Situation selection × Suppression interaction and given that this effect was significant for positive viewing (see below), we present this interaction for negative viewing as illustration in Fig. 7a.

When considering positive trials, the ANOVA performed on the changes in respiratory amplitude showed three significant effects (see Table 4). First, and similarly to what was observed for negative viewing, the test yielded a significant Time × Situation selection interaction (see Fig. 6b). Second, we also found a significant Time × Suppression interaction (see Fig. 6c). Third, and more interestingly, the Situation selection × Suppression interaction was also significant. As shown in Fig. 7b, contrasts for this effect indicated that the combined use of the two strategies markedly decreased respiratory amplitude, at a significantly lower level than when only *Situation selection* was used.

## Discussion

The purpose of this study was to investigate the effect of two emotion regulation strategies used simultaneously on emotional responses. The idea behind this research was that since individuals have a wide range of emotion regulation strategies at their disposal, it is very likely that several strategies will be attempted during the same emotional event. At the time of the writing of this article, a new study was published examining the use of two different strategies (*Reappraisal* and *Rumination)* but focused on their sequential use [82]. Although it did not strictly focus on simultaneous use consequences, it highlights how investigating the interrelation and interaction between several strategies is timely for the field.

Specifically, the present study aimed to observe the combined effect of *Situation selection* and *Emotional suppression* on emotional experience, expressivity and physiological arousal, as compared to when each of these strategies is used alone. To this end, we conducted a within-subject design experiment measuring emotional responses of participants confronted to emotionally loaded stimuli when performing either *Situation selection*, *Emotional suppression,* or both. For single-strategy conditions, we expected to replicate the results obtained in similar studies addressing the impact of these strategies used independently. For the double-strategy condition, we expected at least a summative effect: effects going in opposite directions under single strategy conditions would be canceled, and effects going in the same direction under single-strategy conditions would be enhanced. Our hypotheses were overall confirmed. We discuss below the major effects for each of the single and double conditions.

For *Situation Selection* used alone, and as hypothesized, our results confirmed those of a previous study [22]. Five results can notably be highlighted. First, negative experience decreased for the chosen pictures, as compared to the imposed pictures. Second, no impact of *Situation selection* on positive experience or expressivity was found. Third, performing *Situation selection* before viewing a given picture modified the subsequent cardiovascular response: the decrease in heart rate was stronger, particularly after 3 s of viewing. The heart rate remained lower than when viewing imposed pictures, and this until the end of the viewing period. This decrease could initially be interpreted as a stronger orienting response, reflecting the allocation of more resources to the choice procedure and the stimulus processing when the stimulus is chosen than when it is imposed. However, since this effect persisted until at least 8 s after the beginning of the image viewing, it can be argued that *Situation selection* significantly decreased the cardiovascular reaction to the emotional episode. Fourth, a particular dynamic for the change in skin conductance was found. At the beginning of the period, a higher skin conductance level was observed when the image was chosen, implying that performing a choice triggered an increase in skin conductance level. However, after a few seconds the effect reversed and the skin conductance level dropped at a lower level for chosen pictures than for imposed pictures. This pattern has been interpreted in the past as a successful downregulation of exocrine activity by *Situation selection* [22] and was replicated in the present study. Finally, and also confirming our hypotheses, a greater decrease in respiratory rate under the *Situation selection* condition for both negative and positive viewings was observed. Results also showed a consistent decrease in respiratory amplitude for the same condition. This is an unexpected result. Indeed, the vagal nerve responsible for respiratory variation generally couples rate and amplitude negatively, so that an increase in respiratory rate is associated with a decrease in respiratory amplitude [83, 84]. The present discrepant pattern was already found while examining effects of *Situation selection* and *Suppression* [22, 47]. Interpreting the physiology behind this result, we could state that a decrease in both respiratory rate and amplitude reduces the oxygen intake by lowering the incoming air volume. We could argue that this result represents a process by which oxygen demands are lowered by emotion regulation, thus converging toward reduced stress when emotions are regulated.

Based on the results of previous literature in similar contexts (early reactions to visual emotional stimulations, see introduction section), we hypothesized that *Emotional suppression* would not influence experience, but would strongly impact expressivity and show a stronger decrease in heart rate and respiratory functions. Three lines of results can be discussed. First, negative experience increased when participants performed *Emotional suppression*, compared to when they were not instructed to suppress. This is consistent with studies showing counterproductive effects of *Suppression* on emotional experience [85], although such evidence remains scarce in the literature. Second, and as repeatedly demonstrated, *Emotional suppression* reduced the expressive component of emotion, showing again that participants can successfully implement the requested strategy (see e.g., [35–38, 86, 87]). Third, and also in line with past studies, *Emotional Suppression* triggered a long-lasting decrease in heart rate. We did not find an effect of the strategy on skin conductance level, which is usually a measure that reacts with a rebound to this instruction [36], but we found a positive impact of *Emotional suppression* on respiratory functions. This is similar to results in other studies using similar instructions to implement *Suppression* [47, 48]. This result may suggest that, at least in the first few seconds after image viewing, *Emotional suppression* can have a positive impact on physiological arousal. This is possible since the instructions asking for general *Emotional suppression* (physiological and expressive) also have a physiological component. Thus, participants might have tried to regulate their respiration with a slow and shallower controlled breathing, causing the reduction in rate and amplitude.

Used simultaneously, *Situation selection* and *Emotional suppression* had the following outcomes. First, their respective and opposite effects on negative experience canceled each other out. This shows that performing *Emotional Suppression* is not beneficial to the regulation of experience, congruently with our hypothesis, even when performed together with *Situation Selection*, which usually has a positive impact. With respect to expressivity, and as expected, we found that it was reduced by *Emotional suppression* and unaffected by *Situation selection*. So far, nothing very surprising. However, the picture becomes more interesting when we examine the physiological impact of the combined use of the two emotion regulation strategies. We found that the heart rate deceleration was incrementally potentiated by the combined use of the emotion regulation strategies, and this in both positive and negative contexts. Indeed, using both regulation strategies triggered a greater decrease of heart rate in the last 4 to 5 s of the picture viewing in comparison with the no regulation condition and the single-strategy conditions. Thus, to regulate cardiovascular activity related to emotional stimulation, using both strategies may be more efficient than using only one. With respect to exocrine activity, we found no incremental effect of the double regulation. It seems that, in the case of double regulation, the *Situation selection* effect dominates. Therefore, there is no advantage in using two strategies instead of one for this response. Finally, the results of respiratory activity showed a decrease in oxygen intake for both strategies, with lower respiratory rates and amplitudes. However, this was particularly noticeable in the last 3–3.5 s of picture viewing in the double regulation condition, and particularly for positive viewing. This latter nuance suggests that this mechanism may in fact reflect the regulatory effect, which always appears easier in positive regulation. Indeed, in this context, reducing respiratory activity means letting go to the relaxing or calming effect of the positive viewing (which consisted mostly in low arousing pictures).

On the basis of our results, we could deliberate on the added value of simultaneously using *Situation selection* and *Emotional suppression* to regulate emotions. We found that this added value will greatly depend on the targeted emotional response. The major impact of this double regulation is, in the short term, the successful regulation of physiological responses. Regarding emotional expression, using both strategies will be as effective as using *Emotional suppression* alone, considering *Situation selection* had no effect on expression. Additionally, reducing negative experience is the major asset of *Situation selection*. This effect has been observed repeatedly for *Situation selection* and confers to this strategy a particular value, as there are few strategies that can achieve successful regulation of negative experience with such low investment of resources. However, this effect disappears when *Emotional suppression* is performed together with *Situation selection*. Thus, if the goal of regulation is to cope with negative experience, a combined use of these two strategies cannot be recommended. It may then be preferable to use only *Situation selection*.

These conclusions are interesting when related to past findings about the efficiency of the use of single emotion regulation strategy. For example, a recent study [56] investigated the comparative efficiency of *Distraction* and *Reappraisal* in relationship with trait anxiety. These strategies were applied separately and results showed that although *Distraction* and *Reappraisal* helped regulating emotional experience and expressivity, they had no effect on physiological responses. This is unfortunate because heart and respiration activity are the emotion responses that are specifically impacted by trait anxiety. Our present results could be useful for finding a way to regulate anxiety related responses. The combined use of *Situation selection* and *Emotional suppression* could help people with high trait anxiety for decreasing their cardiac and respiratory activity and lowering their overall physiological arousal. This shows how studies such as the present one, examining combinatory effect of different emotion regulation strategies, could provide solutions for concrete situations in which individual strategies are not effective.

So far, most research has focused on the effect of only one strategy used at a given time. This approach, while informative, does not however reflect what likely happens in everyday situations, when people struggle to downregulate negative emotions, potentially trying everything possible to remain focused on their task or interaction. This is why the next important frontier in the field of emotion regulation research regards how multiple emotion regulation strategies used at the same time will combine themselves and what are their direct collective consequences on emotional responses.

### Limitations

Several limitations can be pointed out regarding this study. First, we focused on right-handed participants who were furthermore all students and generalization of the results is hence limited. More broadly, the next logical step would be to include more characterization of individual differences in order to obtain more detailed conclusions. For example, a recent pilot study in our lab showed that *Situation selection* may be efficient to reduce experience as shown in the present study but only for people who may be high on neuroticism (Big five dimension, unpublished data). In addition, focusing on present symptomatology, like predisposition to anxiety [88] could help to refine the interpretation of the results. Eventually, this would help establishing recommendations on which strategies should be used, based on individual traits and paying close attention to which target emotional response is involved. Second, regarding *Situation selection* implementation, the choices that were given were limited to two options. This did not allow us to disentangle whether the choice was made on the basis of preference for the chosen option, or avoidance of the alternative. The choice given to the participants would deserve to be extended to a less limited and more ecological set of options. Third, we cannot guarantee that only *Emotional suppression* was attempted during our experiment. We are confident in the strategy implementation, since we replicated previous results regarding *Suppression*. However, it is possible that other explicit or even implicit emotion regulation strategies may have been simultaneously implemented, so that our results reflect the efficiency of a blend of strategies, rather than the pure effect of *Suppression*. A related concern on this point is the fact that we could wonder whether imposing a strategy or leaving it self-selected by the participant changes the way it is implemented and therefore its efficiency. This question would deserve particular attention in future studies. A fourth limitation relates to the consideration of broad valence affects (positive and negative) as discussed by dimensional theorists of affect [89–91]. This distinction had the advantage of limiting boundary problems between emotion categories linked to the frequent occurrence of mixed emotions [92]. Yet, this approach cannot provide information on the efficiency of *Situation selection* for particular emotions such as joy, fear or sadness. Further investigations on the type of positive vs. negative affects would provide a better understanding on how to best implement *Situation selection* and *Emotional suppression* in daily situations.

## Conclusions

To our knowledge, this study is the first to investigate the simultaneous effect on emotion responses of two emotion regulation strategies (*Situation selection* and *Emotional suppression*) used at the same time for a single event*.* Our design allowed to compare double-strategy use with each strategy separately and with a condition in which participants were not instructed to regulate. This was done dynamically over the first few seconds of the occurrence of an emotional episode. In conclusion, our study highlights how the combinatorial effect of emotion regulation strategies differs according to the channel to be regulated. With the strategies investigated here, physiological arousal is the emotional response that benefits the most from a double regulation attempt. The combined use of *Situation selection* and *Emotional suppression* is then most beneficial for people who have difficulty regulating the physiological part of their emotions. Such results could improve the recommendation for more functional emotion regulation in daily life.

## Data Availability

The datasets used and/or analyzed during the current study are available from the corresponding author on reasonable request. They also are available from the OFS home repository (https://osf.io/qgzth).

## Abbreviations

ANOVA: Analysis of Variance
ECG: Electrocardiography
EMG: Electromyography
GAPED: Geneva Affective Picture Database
IAPS: International Affective Picture System
SEM: Standard Error of the Mean
SF-12: 12-Item Short-Form Health Survey

## References

[1] Gross JJ, Feldman Barrett L (2011). Emotion generation and emotion regulation: one or two depends on your point of view. Emot Rev.

[2] Gross JJ, Richards JM, John OP, Snyder DK, Simpson JE, Hughes JN (2006). Emotion regulation in everyday life. Emotion regulation in couples and families: pathways to dysfunction and health.

[3] Keltner D, Gross JJ (1999). Functional accounts of emotions. Cognit Emot.

[4] Tomkins S, Scherer K, Ekman P (1984). Affect theory. Approaches to emotion.

[5] Gross JJ (1998). The emerging field of emotion regulation: an integrative review. Rev Gen Psychol.

[6] Gross JJ, John OP (2003). Individual differences in two emotion regulation processes: implication for affect, relationships, and well-being. J Pers Soc Psychol.

[7] Gross JJ, Muñoz RF (1995). Emotion regulation and mental health. Clin Psychol Sci Pract.

[8] Eisenberg N, Fabes RA, Guthrie IK, Reiser M (2000). Dispositional emotionality and regulation: their role in predicting quality of social functioning. J Pers Soc Psychol.

[9] Hayes SC, Wilson KG, Gifford EV, Follette VM, Strosahl K (1996). Experiential avoidance and behavioral disorders: a functional dimensional approach to diagnosis and treatment. J Consult Clin Psychol.

[10] Campbell-Sills L, Barlow DH, Gross JJ (2007). Incorporating emotion regulation into conceptualizations and treatments of anxiety and mood disorders. Handbook of emotion regulation.

[11] Mennin DS, Heimberg RG, Turk CL, Fresco DM (2002). Applying an emotion regulation framework to integrative approaches to generalized anxiety disorder. Clin Psychol Sci Pract.

[12] Mennin DS, Heimberg RG, Turk CL, Fresco DM (2005). Emotion regulation deficits as a key feature of generalized anxiety disorder: testing a theoretical model. Behav Res Ther.

[13] Tull MT, Roemer L (2007). Emotion regulation difficulties associated with the experience of uncued panic attacks: evidence of experiential avoidance, emotional nonacceptance, and decreased emotional clarity. Behav Ther.

[14] Cloitre M, Stovall-McClough K, Miranda R, Chemtob CM (2004). Therapeutic alliance, negative mood regulation, and treatment outcome in child abuse-related posttraumatic stress disorder. J Consult Clin Psychol.

[15] Glenn CR, Klonsky ED (2009). Emotion dysregulation as a core feature of borderline personality disorder. J Personal Disord.

[16] Henry JD, Green MJ, de Lucia A, Restuccia C, McDonald S, O'Donnell M (2007). Emotion dysregulation in schizophrenia: reduced amplification of emotional expression is associated with emotional blunting. Schizophr Res.

[17] Westermann S, Boden MT, Gross JJ, Lincoln TM (2013). Maladaptive cognitive emotion regulation prospectively predicts subclinical paranoia. Cogn Ther Res.

[18] Gross JJ (2001). Emotion regulation in adulthood: timing is everything. Curr Dir Psychol Sci.

[19] Gross JJ (2007). Handbook of emotion regulation.

[20] Gross JJ, Thompson RA, Gross JJ (2007). Emotion regulation: conceptual foundations. Handbook of emotion regulation.

[21] Jazaieri H, McGonigal K, Lee IA, Jinpa T, Doty JR, Gross JJ (2017). Altering the trajectory of affect and affect regulation: the impact of compassion training. Mindfulness..

[22] Thuillard S, Dan-Glauser E (2017). The regulatory effect of choice in situation selection reduces experiential, exocrine and respiratory arousal for negative emotional stimulations. Sci Rep.

[23] Sands M, Isaacowitz DM (2016). Situation selection across adulthood: the role of arousal. Cognit Emot.

[24] Livingstone KM, Isaacowitz DM (2015). Situation selection and modification for emotion regulation in younger and older adults. Soc Psychol Personal Sci.

[25] Aldao A, Nolen-Hoeksema S (2013). One versus many: capturing the use of multiple emotion regulation strategies in response to an emotion-eliciting stimulus. Cognit Emot.

[26] Szasz PL, Coman M, Curtiss J, Carpenter JK, Hofmann SG (2018). Use of multiple regulation strategies in spontaneous emotion regulation. Int J Cogn Ther.

[27] Opitz PC, Cavanagh SR, Urry HL (2015). Uninstructed emotion regulation choice in four studies of cognitive reappraisal. Personal Individ Differ.

[28] Bonanno GA, Burton CL (2013). Regulatory flexibility:an individual differences perspective on coping and emotion regulation. Perspect Psychol Sci.

[29] Aldao A, Nolen-Hoeksema S (2012). When are adaptive strategies most predictive of psychopathology?. J Abnorm Psychol.

[30] Lee DJ, Witte TK, Weathers FW, Davis MT (2015). Emotion regulation strategy use and posttraumatic stress disorder: associations between multiple strategies and specific symptom clusters. J Psychopathol Behav Assess.

[31] Otterpohl N, Schwinger M, Wild E (2016). Exploring the interplay of adaptive and maladaptive strategies: prevalence and functionality of anger regulation profiles in early adolescence. J Early Adolesc.

[32] Birk JL, Bonanno GA (2016). When to throw the switch: the Adaptiveness of modifying emotion regulation strategies based on affective and physiological feedback. Emotion..

[33] Webb TL, Miles E, Sheeran P (2012). Dealing with feeling: a meta-analysis of the effectiveness of strategies derived from the process model of emotion regulation. Psychol Bull.

[34] Cutuli D (2014). Cognitive reappraisal and expressive suppression strategies role in the emotion regulation: an overview on their modulatory effects and neural correlates. Front Syst Neurosci.

[35] Gross JJ (1998). Antecedent- and response-focused emotion regulation: divergent consequences for experience, expression, and physiology. J Pers Soc Psychol.

[36] Gross JJ, Levenson RW (1993). Emotional suppression: physiology, self-report, and expressive behavior. J Pers Soc Psychol.

[37] Gross JJ, Levenson RW (1997). Hiding feelings: the acute effects of inhibiting negative and positive emotion. J Abnorm Psychol.

[38] Roberts N, Levenson RW, Gross JJ (2008). Cardiovascular costs of emotion suppression cross ethnic lines. Int J Psychophysiol.

[39] Dunn BD, Billotti D, Murphy V, Dalgleish T (2009). The consequences of effortful emotion regulation when processing distressing material: a comparison of suppression and acceptance. Behav Res Ther.

[40] Goldin PR, McRae K, Ramel W, Gross JJ (2008). The neural bases of emotion regulation: reappraisal and suppression of negative emotion. Biol Psychiatry.

[41] Boland M, Papa A, del Carlo RE (2019). Trait negative affect moderates the effects of expressive versus experiential emotion suppression. Personal Individ Differ.

[42] Lemaire M, El-Hage W, Frangou S (2014). Reappraising suppression: subjective and physiological correlates of experiential suppression in healthy adults. Front Psychol..

[43] Geisler FCM, Schroeder-Abe M (2015). Is emotion suppression beneficial or harmful? It depends on self-regulatory strength. Motiv Emot.

[44] Gracanin A, Kardum I, Hudek-Knezevic J (2017). Parasympathetic concomitants of habitual, spontaneous, and instructed emotional suppression. J Psychophysiol.

[45] Olatunji BO, Berg HE, Zhao Z (2017). Emotion regulation of fear and disgust: differential effects of reappraisal and suppression. Cognit Emot.

[46] Lee E-J, Kim H-C, Park JH, Lee J (2018). The moderating effect of gender in the relationship between emotional suppression and blood pressure. J Korean Data Anal Soc.

[47] Dan-Glauser ES, Gross JJ (2011). The temporal dynamics of two response-focused forms of emotion regulation: experiential, expressive, and autonomic consequences. Psychophysiology..

[48] Dan-Glauser ES, Gross JJ (2015). The temporal dynamics of emotional acceptance: experience, expression, and physiology. Biol Psychol.

[49] Lang PJ, Greenwald MK, Bradley MM, Hamm AO (1993). Looking at pictures: affective, facial, visceral, and behavioral reactions. Psychophysiology..

[50] Siedlecka E, Denson TF (2019). Experimental methods for inducing basic emotions: a qualitative review. Emot Rev.

[51] Bradley MM, Lang PJ (2000). Measuring emotion: behavior, feeling, and physiology. Cognitive neuroscience of emotion. Series in affective science.

[52] Lang PJ, Bradley MM, Cuthbert BN (1998). Emotion, motivation, and anxiety: brain mechanisms and psychophysiology. Biol Psychiatry.

[53] Cohen J (1988). Statistical power analysis for the behavioral sciences.

[54] Ware JE, Kosinski M, Keller SD (1996). A 12-item short-form health survey: construction of scales and preliminary tests of reliability and validity. Med Care.

[55] Reuter-Lorenz P, Givis R, Moscovitch M (1983). Hemispheric specialization and the perception of emotion: evidence from right-handers and from inverted and non-inverted left-handers. Neuropsychologia..

[56] Bourne VJ (2008). Examining the relationship between degree of handedness and degree of cerebral lateralization for processing facial emotion. Neuropsychology..

[57] Oldfield RC (1971). The assessment and analysis of handedness: the Edinburgh inventory. Neuropsychologia..

[58] Stein NL, Trabasso T, Liwag M, Lewis M, Haviland JM (1993). The representation and organization of emotional experience: unfolding the emotion episode. Handbook of emotions.

[59] Esslen M, Pascual-Marqui RD, Hell D, Kochi K, Lehmann D (2004). Brain areas and time course of emotional processing. NeuroImage..

[60] Bradley MM, Lang PJ, Lane RD, Nadel L (2000). Measuring emotion: Behavior, feeling, and physiology. Cognitive neuroscience of emotion.

[61] Codispoti M, Bradley MM, Lang PJ (2001). Affective reactions to briefly presented pictures. Psychophysiology..

[62] Dan-Glauser ES, Scherer KR (2011). The Geneva affective picture database (GAPED): a new 730-picture database focusing on valence and normative significance. Behav Res Methods.

[63] Lang PJ, Bradley MM, Cuthbert BN. International Affective Picture System (IAPS): instruction manual and affective ratings. Technical Report A-4, Center for Research in Psychophysiology: University of Florida.; 1999.

[64] Kring AM, Gordon AH (1998). Sex differences in emotion: expression, experience, and physiology. J Pers Soc Psychol.

[65] Matsumoto D, Nezlek JB, Koopmann B (2007). Evidence for universality in phenomenological emotion response system coherence. Emotion..

[66] Mauss IB, Levenson RW, McCarter L, Wilhelm FH, Gross J (2005). The tie that binds? Coherence among emotion experience, behavior, and physiology. Emotion..

[67] Larsen JT, Norris CJ, Cacioppo JT (2003). Effects of positive and negative affect on electromyographic activity over zygomaticus major and corrugator supercilii. Psychophysiology..

[68] Frank MG, Ekman P, Friesen WV (1993). Behavioral markers and recognizability of the smile of enjoyment. J Pers Soc Psychol.

[69] Fridlund AJ, Cacioppo JT (1986). Guidelines for human electromyographic research. Psychophysiology..

[70] Van Boxtel A (2010). Facial EMG as a tool for inferring affective states. Proceedings of measuring behavior.

[71] de Wied M, van Boxtel A, Zaalberg R, Goudena PP, Matthys W (2006). Facial EMG responses to dynamic emotional facial expressions in boys with disruptive behavior disorders. J Psychiatr Res.

[72] Lang PJ, Bradley MM, Cuthbert BN, Lang PJ, Simons RF, Balaban MT (1997). Motivated attention: Affect, activation, and action. Attention and orienting: Sensory and motivational processes.

[73] Palomba D, Angrilli A, Mini A (1997). Visual evoked potentials, heart rate responses and memory to emotional pictorial stimuli. Int J Psychophysiol.

[74] VanOyen WC, Vrana SR (1995). Psychophysiological responses as indices of affective dimensions. Psychophysiology..

[75] Kensinger EA, Schacter DL (2006). Processing emotional pictures and words: effects of valence and arousal. Cogn Affect Behav Neurosci.

[76] Dolcos F, LaBar KS, Cabeza R (2004). Dissociable effects of arousal and valence on prefrontal activity indexing emotional evaluation and subsequent memory: an event-related fMRI study. Neuroimage..

[77] Winton WM, Putnam LE, Krauss RM (1984). Facial and autonomic manifestations of the dimensional structure of emotion. J Exp Soc Psychol.

[78] Hubert W, de Jong-Meyer R (1991). Autonomic, neuroendocrine, and subjective response to emotion-inducing film stimuli. Int J Psychophysiol.

[79] Mak AK, Hu Z-G, Zhang JX, Xiao Z-W, Lee TM (2009). Neural correlates of regulation of positive and negative emotions: an fMRI study. Neurosci Lett.

[80] Kim SH, Hamann S (2007). Neural correlates of positive and negative emotion regulation. J Cogn Neurosci.

[81] Landsheer JA, van den Wittenboer G, Maassen GH (2006). Additive and multiplicative effects in a fixed 2×2 design using ANOVA can be difficult to differentiate: demonstration and mathematical reasons. Soc Sci Res.

[82] Peuters C, Kalokerinos EK, Pe ML, Kuppens P (2019). Sequential effects of reappraisal and rumination on anger during recall of an anger-provoking event. PLoS One.

[83] Porges SW, Raskin DC (1969). Respiratory and heart rate components of attention. J Exp Psychol.

[84] Parhizgar F, Nugent K, Raj R (2011). Obstructive sleep apnea and respiratory complications associated with vagus nerve stimulators. J Clin Sleep Med.

[85] Campbell-Sills L, Barlow DH, Brown TA, Hofmann SG (2006). Acceptability and suppression of negative emotion in anxiety and mood disorders. Emotion..

[86] Jackson D, Malmstadt J, Larson C, Davidson R (2000). Suppression and enhancement of emotional responses to unpleasant pictures. Psychophysiology..

[87] Richards JM, Gross JJ (1999). Composure at any cost? The cognitive consequences of emotion suppression. Personal Soc Psychol Bull.

[88] Efinger L, Thuillard S, Dan-Glauser ES (2019). Distraction and reappraisal efficiency on immediate negative emotional responses: role of trait anxiety. Anxiety Stress Coping..

[89] Posner J, Russell JA, Peterson BS (2005). The circumplex model of affect: an integrative approach to affective neuroscience, cognitive development, and psychopathology. Dev Psychopathol.

[90] Barrett LF, Mesquita B, Ochsner KN, Gross JJ (2007). The experience of emotion. Annual review of psychology. Annu Rev Psychol.

[91] Russell JA (1980). A circumplex model of affect. J Pers Soc Psychol.

[92] Ellsworth PC, Scherer KR, Davidson RJ, Scherer KR, Goldsmith HH (2003). Appraisal processes in emotion. Handbook of affective sciences.

